# Anthropogenically-Mediated Density Dependence in a Declining Farmland Bird

**DOI:** 10.1371/journal.pone.0139492

**Published:** 2015-10-02

**Authors:** Jenny C. Dunn, Keith C. Hamer, Tim G. Benton

**Affiliations:** School of Biology, Irene Manton Building, University of Leeds, Leeds, LS2 9JT, United Kingdom; Hungarian Academy of Sciences, HUNGARY

## Abstract

Land management intrinsically influences the distribution of animals and can consequently alter the potential for density-dependent processes to act within populations. For declining species, high densities of breeding territories are typically considered to represent productive populations. However, as density-dependent effects of food limitation or predator pressure may occur (especially when species are dependent upon separate nesting and foraging habitats), high territory density may limit per-capita productivity. Here, we use a declining but widespread European farmland bird, the yellowhammer *Emberiza citrinella* L., as a model system to test whether higher territory densities result in lower fledging success, parental provisioning rates or nestling growth rates compared to lower densities. Organic landscapes held higher territory densities, but nests on organic farms fledged fewer nestlings, translating to a 5 times higher rate of population shrinkage on organic farms compared to conventional. In addition, when parental provisioning behaviour was not restricted by predation risk (i.e., at times of low corvid activity), nestling provisioning rates were higher at lower territory densities, resulting in a much greater increase in nestling mass in low density areas, suggesting that food limitation occurred at high densities. These findings in turn suggest an ecological trap, whereby preferred nesting habitat does not provide sufficient food for rearing nestlings at high population density, creating a population sink. Habitat management for farmland birds should focus not simply on creating a high nesting density, but also on ensuring heterogeneous habitats to provide food resources in close proximity to nesting birds, even if this occurs through potentially restricting overall nest density but increasing population-level breeding success.

## Introduction

Population densities of animal species are influenced by both density-dependent and density-independent processes [1]; however, the mechanisms behind many of these processes are poorly understood [2–4]. Land management intrinsically influences the distribution of animals and can consequently alter the potential for density-dependent processes to act within populations. Knowledge of the mechanisms of population regulation is key to understanding population dynamics [5] and how species may respond to environmental change [2,3,6].

Animals inhabiting intensively managed habitats frequently face resource shortages that may influence individuals’ growth, survival and reproduction (e.g. [7]). High densities of breeding animals usually represent healthy populations with higher recruitment rates [8], but this may be confounded where animals are dependent upon separate breeding and foraging habitats, or where high densities may preferentially attract predators. In this former case, animals selecting breeding sites based on limited breeding habitat may either be constrained in their foraging ranges and thus food-limited, or unable to assess foraging habitat quality at the time of settlement due to subsequent anthropogenic management. If food availability is universally limiting, then high breeding densities may represent areas of increased competition for food resources [9,10] where the energetic requirements of raising offspring are increased. In addition to increased competition for food, higher breeding densities may also attract an increased predation risk, through predators being attracted to areas with higher prey densities [11–13]. Consequently, areas of high breeding density may in fact represent population sinks [14]. Sink habitats that are selected preferentially over source habitats have been termed “attractive sinks” [15] and represent an ecological trap where the fitness of an animal in its preferred habitat is lower than in less preferred habitats [16].

Ecological traps are most common in areas that receive high levels of anthropogenic disturbance [8,17,18]. For example, in agricultural habitats both vegetation structure and the fauna it supports can change dramatically following the application of pesticides, herbicides or following physical management such as the mowing of vegetated margins. Many farmland species have declined significantly since the onset of agricultural intensification (e.g. [19]) and whilst areas of high territory density are thought to reflect high quality habitat, to our knowledge the possibility of an ecological trap occurring in the context of high territory densities leading to low productivity within such an anthropogenically influenced environment has not been examined. Declining populations may be increasingly susceptible to ecological traps as reduced competition within sink habitat removes individuals from non-preferred source habitat [20,21].

Here, we use a declining but widespread European farmland bird, the yellowhammer *Emberiza citrinella*, as a model system: this socially monogamous, resident species is territorial over nest sites but not foraging sites [22]. Territory density in this species is associated with the availability of suitable nesting habitat such as hedgerows and ditches, and yellowhammers show preference for short wide hedgerows with no trees [23,24]. The removal of these habitat features is thought to have contributed to population declines [25] and consequently the species is thought to be limited by the availability of suitable nesting habitat [26]. Yellowhammers typically lay 3–4 eggs per clutch, with 2–3 broods per year [27] and adults are granivorous, although they rely on invertebrates to provision nestlings. Nestling food availability can limit productivity at a local scale [7] and nest predator activity can limit parental provisioning [28]. We examine population densities across a range of habitat types, and two farm management practices (organic and conventional) to test three hypotheses regarding territory density and breeding success.

First, we test whether higher territory densities result in lower fledging success. We predict that birds nesting at higher territory densities will have a higher failure rate due to density-dependent nest predation, a higher chance of brood reduction due to competition for limited food resources, and consequently a lower fledgling number per successful nesting attempt, reducing their overall population contribution. Second, we test whether birds nesting in areas of higher territory density exhibit lower parental provisioning rates due to competition for food. We expect to find this especially within farmland environments because anthropogenic changes (application of herbicides and pesticides, mowing of margins, etc.) will make invertebrate availability during nestling rearing unpredictable at the time of territory establishment. We expect to find an interaction with corvid activity, as adult birds in this system reduce provisioning rates at times of high corvid activity [28] and thus we expect to see a negative relationship between territory density and provisioning rate only when provisioning rate is not otherwise limited (i.e. at times of low corvid activity). Finally, we test whether territory density impacts upon nestling mass, expecting that nestlings in areas of high territory density would show reduced growth rates as a result of lower provisioning rates or increased competition for high quality food. We find evidence for high nesting densities representing population sinks, not sources.

## Materials and Methods

### Sites

Fieldwork was carried out during May–July 2007 and 2008 on 18 farms in Wiltshire, Hampshire and Gloucestershire, UK. Farms consisted of pairs of organic and conventional farms, matched according to soil type, size, geography and ratio of arable to livestock, within either organic (where on average 17.2% of land cover within a 10 x 10 km square was farmed organically) or conventional landscapes (where on average 1.4% of equivalent land cover was farmed organically; see [29] for further details). All further references to management refer to either farm-scale management (farm management) or landscape-scale management (landscape management). Sixteen farms were surveyed in each year, with two farms being replaced in 2008 due to changing farm management practices [28]. Territory data were collected from 16 additional farms during 2008, extending the study area into West Sussex, Staffordshire, Leicestershire, Derbyshire and Shropshire, [29]. Additional data on nest success were collected during May–August 2006 from three farms in North Yorkshire, UK: we did not collect corvid activity or abundance, or comparable invertebrate abundance data from these sites.

### Territory density and habitat variables

Farms were visited on a minimum of three occasions during each breeding season and territories mapped according to the location of singing males. Each male was observed for a minimum of one hour approximately every two weeks during the first half of the breeding season to differentiate between close neighbours singing at different times, and the same bird using more than one song-post within a larger territory. Where farms were too large for adequate coverage within a morning, only a subsection of the farm was surveyed: this subsection was determined both by ease of access and by the location of focal cereal fields for a concurrent project using the same sites [29]. No territory surveys were carried out under wet or windy conditions as this reduced singing behaviour and made locating and following birds more difficult. Boundary vegetation was identified for each length of field boundary between intersections to identify boundary habitats associated with territory density and classified according to Table 1 to encompass the habitats within which the majority of nests were located [30]; if vegetation changed within this unit the point at which this change occurred was estimated.

**Table 1:** Classification of boundary habitat vegetation.

Habitat	Definition
Hedge	Hedgerow less than 3m in height with <10% canopy cover from trees over 3m in height
Hedge with 10–50% trees	Hedgerow less than 3m in height with 10–50% canopy cover from trees over 3m in height
Hedge with >50% trees	Hedgerow with >50% canopy cover from trees over 3m in height; also includes tree lines with no hedgerow vegetation
Fence with vegetation	Fence or other boundary (except hedgerow) adjoined by >1m width of dense herbaceous vegetation
Gappy hedge	Hedgerow with >20% gaps along length; also includes rows of solitary bushes
Fence or wall	Fence or other boundary (except hedgerow) adjoined by <1m width of dense herbaceous vegetation.

Territories were mapped in ArcGIS based on the centre of the territory being the song-post where each male was observed most frequently. Two measures of territory density were calculated: 1) The nearest neighbour distance (NND), defined as the distance to the centre of the nearest neighbouring territory (to a maximum limit of 1 km where no neighbour was present within this distance); and 2) the average distance to the three nearest neighbours (NTND), using the same criteria as defined in 1). Boundary habitats (Table 1) were mapped in ArcGIS and the total length of each boundary habitat was calculated on each farm.

### Nest, nestling and behavioural data

Nests were located using the methodology described by Bradbury *et al*. [25]: once located, the height of the nest above ground, and vegetation within which the nest was built were recorded. Nest concealment was judged from photographs taken from a distance of 2 m from the nest, from the angle at which the nest was most obvious without disturbance of vegetation. Concealment was scored on a categorical scale from 1 (poorly concealed) to 5 (well concealed); photos were scored blind to nest identity and outcome. Nests were monitored and nestlings measured as described in [28,30]: in brief, nests were monitored at maximum intervals of 3 days and nestlings were measured on two occasions between two and seven days of age, the period of linear growth for this species [31]. Where a nest was located at the nestling stage, nestling age was estimated through comparison of feather tract development with nestlings of known age. Where a nest contained nestlings at 7 days and the nest remained intact but was empty at 10 days (making predation of nestlings immediately prior to fledging unlikely), the nestlings was deemed to have fledged successfully. If the nest was damaged, we assumed the nestlings had not fledged successfully. We monitored a maximum of one nest per territory so as to avoid confounding effects of parental or territory quality within our analyses.

Observations of adult foraging behaviour were carried out when nestlings were between 2 and 7 days old: provisioning watches were carried out and provisioning rate calculated as detailed in [28] to provide the number of complete foraging trips per nest per hour.

### Predator and prey abundance

Corvids [magpies *Pica pica* (Linnaeus), carrion crows *Corvus corone* (Linnaeus), rooks *Corvus frugilegus* (Linneaus) and jackdaws *Corvus monedula* (Linneaus)] are the main nest predators of the yellowhammer in the UK [25]. Whilst nest predation due to other predators (e.g. least weasels *Mustela nivalis* (Linnaeus), stoats *Mustela erminea* (Linneaus), brown rats *Rattus norvegicus* (Berkenhout)) does occur within this system, yellowhammers have previously been found to adjust nestling provisioning behaviour in response to corvid activity [28] and thus only corvids are considered further here. As corvid activity exhibits marked temporal variation [32], we assessed corvid abundance at both the territory scale and the farm scale. Territory scale corvid abundance, hereafter termed ‘corvid activity’, was assessed using 20 min point count surveys carried out immediately prior to each provisioning watch to assess the total number of corvids within 100m of the nest. Farm-scale corvid abundance, hereafter termed ‘corvid abundance’, was assessed using two 1 km transects walked on each farm on three separate occasions between May and July during both 2007 and 2008, according to standard methodology [33]. The total number of corvids seen and heard within 250m either side of the transect path was averaged across all three visits to provide a farm scale measure of corvid abundance per farm per year.

The abundance of invertebrates greater than 2 mm in length, within taxonomic groups known to be important in the diet of yellowhammer nestlings [34,35] was assessed at the level of the individual territory, as invertebrate abundance shows marked temporal and spatial variation (e.g. [36]). Field margins are a favoured foraging habitat of yellowhammers, and the majority of foraging trips are within 200m of the nest with 60% within 100m [37]. Thus, our assessment of invertebrate abundance was designed to obtain a comparable measure of invertebrate abundance within potential foraging habitat. We describe detailed sampling methodology elsewhere [28]. In order to accurately associate provisioning rate with invertebrate abundance (which varies temporally), we collected invertebrate samples immediately following provisioning watches at each nest. Further details of corvid activity and abundance and invertebrate abundance data collections are described elsewhere [28]; these data were collected during 2007 and 2008 only to test for interactions between territory density and resource availability.

### Statistical analysis

Statistical analyses were carried out in R version 3.0.2 for Mac [38]. We used the ‘dredge’ function within the *MuMIn* package [39] to rank candidate models using second-order Akaike’s Information Criteria (AICc) [40]. AICc selects models with the maximum goodness of fit while retaining the minimum number of variables. All models were run twice, substituting NTND for NND in the second model. NTND and NND were highly correlated (Pearson’s product-moment correlation = 0.95, *P*<0.001), and the replacement of NND by NTND did not alter the direction or magnitude of effects. Thus, we describe only the methods and results for models including NND.

#### Model selection and interpretation

For all subsequent analyses, we constructed a global model, selected the best fitting models using AICc comparisons, and averaged all models with AICc < 2 to provide parameter estimates and confidence intervals [40]. Terms retained in at least one of the top models were considered to influence the fit of the model, but only terms with confidence intervals not overlapping zero were considered to significantly influence the response variable. We calculated R^2^ values from the top model for each analysis, prior to averaging, by calculating the R^2^ metric proposed by Nakagawa & Schielzeth [41] and extended by Johnson [42]. We present both the marginal (fixed effects only) and conditional (fixed and random effects) R^2^ values to provide measures of model performance in the legend to each model table.

#### Number of territories

First, we examined factors influencing the distribution of territories, to confirm that habitat influenced the number of territories at the within-farm scale and that this varied between farm managements. We constructed a generalized linear mixed effects model (GLMM) with territory number per farm per year as the response variable and assumed a Poisson distribution for territory number. Terms included in the global model include the length of all boundary features (Table 1), year, geographic location (a six-level term denoting to which geographic ‘cluster’ each farm belonged), and an interaction between farm management and landscape management, with all lengths log + 1 transformed in order to meet model assumptions. Our first model indicated that hedgerow length and the length of fence with vegetation significantly influenced territory number. Next, we then examined whether farm- or landscape management influenced variation in territory number, hedgerow length and the length of fence with vegetation at the between-farm scale, in order to assess whether the number of territories was anthropogenically influenced. To do this, we constructed three univariate generalized linear models (GLM) and tested the influence of farm ID on the subset of farms surveyed in both years (n = 10).

#### Nest outcome

Second, we investigated whether territory density (for which we subsequently use NND as a proxy) influenced the probability of total or partial nest failure (designated as a binomial response of brood reduced or brood not reduced), or the number of fledglings. We carried out four separate analyses, splitting the nesting period into egg and nestling stages, and also investigating brood reduction and the number of fledglings from successful nests. With the exception of the fledgling model, we controlled for the length of time for which a nest was monitored at each stage by using an extension of the Mayfield method [43] adapted for use in GLMs to allow for the inclusion of covariates [43–45], by including both the binomial outcome (0 as success, 1 as failure) and the number of exposure days at the relevant stage as a two-level response variable. Total exposure days were calculated as the number of days between finding the nest and the mid point between the two final visits, using the “Last Active-B” approach recommended by Manolis, Andersen & Cuthbert [46]. We split exposure days into egg- and nestling stage either from observations of hatch date or by estimating hatch date from assessment of nestling age by comparison of feather tract development with that of known age nestlings. For the fledgling model we used the *ordinal* package [47] in R for the examination of ordinal data within a mixed model framework. We excluded all nests that failed to fledge any young, and used the number of fledglings as the response variable within a cumulative link mixed model [48]. Terms included in the global models were day found (Julian day as both linear and quadratic terms), nest height, concealment, farm management, NND, and either clutch size or brood size upon hatching. For the nestling stage, brood reduction and fledgling models we also included an interaction between parental provisioning rate and nest concealment in the global model to allow for the possibility that vegetation cover around the nest can interact with parental behaviour to mediate nest survival (e.g. [47]). We did not include predator or food availability variables in these models as these variables were not assessed during 2006 (n = 20 nests), and we suspected that vegetation cover may be a good surrogate for corvid predation risk as corvids predate more visible nests (e.g. [48]). All models contained Farm ID as a random effect to control for spatial autocorrelation; nestling stage, brood reduction and fledgling models also contained Nest ID as a nested random term to allow for repeated measures of parental provisioning rate per nest.

#### Parental provisioning rate

Next we determined whether territory density influenced parental provisioning rate. To test this, we constructed a linear mixed-effects model with provisioning rate as the response variable and nestling age as a fixed term in the model, with Nest ID as a random effect to control for multiple measures of provisioning rate for the same nest, and spatial autocorrelation. Terms included in the global model were time of day and temperature, as both linear and quadratic terms, year, farm management, NND, invertebrate abundance, brood size and corvid activity. We expected that impacts of territory density on reproductive variables would also be dependent on resource requirement and availability and thus we also included two-way interactions between territory density and each of invertebrate abundance, corvid activity and brood size and farm management in the global model.

#### Nestling mass gain

Finally, we examined whether increased territory density decreased nestling growth, as expected if food resources were limiting. We assessed this by examining the change in nestling mass between two time points, controlling for the non-independence of multiple nestlings within the same nest, as well as spatial autocorrelation, by designating Nest ID as a random term included within all models. This model designated 2^nd^ mass measurement as the response variable, with 1^st^ mass, hours between measurements, and age and time of second measurement as fixed terms in the model. Additional terms tested in the global model were temperature (both linear and quadratic terms), year, NND, invertebrate abundance, brood size, corvid abundance, provisioning rate and farm management. We also tested whether impacts of NND on reproductive variables would also be dependent on resource requirement and availability and thus we examined two-way interactions between NND and each of invertebrate abundance, corvid abundance, provisioning rate and brood size and farm management.

### Ethics statement

Nests were monitored and nestlings measured under license from the British Trust for Ornithology. Sites were privately owned farmland, and work was carried out with permission from landowners.

## Results

### Number of territories

Across 38 farms we identified 213 yellowhammer territories. The number of territories was positively influenced by the length of hedgerow (coefficient ± SE: 0.39 ± 0.10) and the length of fence with vegetation (coefficient ± SE: 0.07 ± 0.03), and differed between years (Table 2), with significantly fewer territories in 2008 than in 2007 (predicted mean ± SE from final model with median values of all other variables within an organic landscape (Table 2); 2007: 3.19 ± 0.30 territories; 2008: 2.22 ± 0.19 territories). The number of territories was also higher in an organic landscape, although farm management was not retained in the final model and thus did not influence territory number (Table 2).

**Table 2:** Results of a GLMM examining the number of yellowhammer territories per farm.

Variable	No. models	Estimate	SE	Lower CI	Upper CI
Intercept	5	-1.231	0.910	-3.014	0.552
**Hedgerow**	**5**	**0.394**	**0.104**	**0.189**	**0.598**
**Year (2008)**	**5**	**-0.748**	**0.179**	**-1.099**	**-0.397**
**Fence with vegetation**	**4**	**0.065**	**0.028**	**0.010**	**0.119**
**Landscape management (organic)**	**3**	**0.364**	**0.178**	**0.014**	**0.713**
10–50% trees	1	0.043	0.032	-0.019	0.105
>50% trees	2	-0.056	0.037	-0.130	0.017

Results presented are those from averaging the five top models where ∆AIC < 2; marginal R^2^ = 0.48; conditional R^2^ = 0.77. Farm ID is included as a random factor within the model. In all tables, terms considered to influence the response variable where confidence intervals do not overlap zero are highlighted in bold.

The length of hedgerow differed between farms (F_10,19_ = 3.89, *P* = 0.02), and the length of fence with vegetation differed marginally between farms (F_10,19_ = 2.91, *P* = 0.056). The number of territories also differed between farms (Dev_10,19_ = -32.43, *P* < 0.001).

### Nest outcome

Nest survival was assessed for 48 nests on 17 farms, of which 22 nests were located at the egg stage, 39 nests on 15 farms reached the nestling stage, and 25 nests on 14 farms successfully fledged young. Mean clutch and initial brood sizes ± SE were 3.32 ± 0.15 eggs and 2.91 ± 0.14 nestlings respectively. At the egg stage, only clutch size remained in the final model predicting failure rate, and confidence intervals overlapped zero (Table 3). At the nestling stage, daily failure probability was influenced by an interaction between provisioning rate and nest concealment (Table 4) whereby well concealed nests had a consistently low failure rate regardless of provisioning rate, but failure rates of poorly concealed nests increased with increasing provisioning rate. However, predicted confidence intervals overlapped each other and thus we do not discuss this result further. Of 34 nests reaching the nestling stage where initial brood size was known, 8 nests (24%) suffered partial brood reduction, with brood reduction more likely to occur in poorly concealed nests (Table 5), but no evidence of an influence of territory density. Whilst NND was retained in the final model predicting the number of fledglings, confidence intervals overlapped zero suggesting no influence (Table 6). However, the number of fledglings per nest increased with increasing nest concealment (mean ± SE from raw data; concealment 3: 1.83 ± 0.32; concealment 4: 3.33 ± 0.16; concealment 5: 3.17 ± 0.15 fledglings per nest), and was lower on organic farms when compared to conventionally managed farms (mean ± SE from raw data; organic: 2.42 ± 0.26 fledglings per nest; conventional: 3.19 ± 0.14 fledglings per nest; Table 6).

**Table 3:** Averaged model estimates and 95% confidence intervals from the top models predicting egg stage failure (marginal R^2^ = 0.22; conditional R^2^ = 0.22); b); c) and d).

	No. models	Estimate	SE	Lower CI	Upper CI
Intercept	2	-8.053	2.959	-13.853	-2.253
Clutch size	1	1.363	0.792	-0.190	2.916

**Table 4:** Averaged model estimates and 95% confidence intervals from the top models predicting nestling stage failure (marginal R^2^ = 0.56; conditional R^2^ = 0.69).

	No. models	Estimate	SE	Lower CI	Upper CI
Intercept	9	-2.228	3.211	-8.521	4.065
Day found^2^	2	0.001	0.001	-0.001	0.001
Brood size	1	-0.688	0.652	-1.966	0.589
NND	5	-0.007	0.006	-0.190	0.005
**Provisioning rate**	**2**	**1.075**	**0.518**	**0.060**	**2.090**
Concealment	6	-0.565	0.877	-2.284	1.155
**Provisioning rate x Concealment**	**2**	**-0.274**	**0.139**	**-0.547**	**-0.002**

**Table 5:** Averaged model estimates and 95% confidence intervals from the top models predicting brood reduction (marginal R^2^ = 0.53; conditional R^2^ = 0.53).

	No. models	Estimate	SE	Lower CI	Upper CI
Intercept	3	-3.319	2.029	-7.294	0.657
**Day found**	**1**	**0.044**	**0.016**	**0.013**	**0.075**
**Day found** ^**2**^	**2**	**0.001**	**0.001**	**0.001**	**0.001**
Brood size	1	0.699	0.547	-0.373	1.770
Farm management	3	1.910	1.039	-0.126	3.946
**Concealment**	**3**	**-1.036**	**0.482**	**-1.980**	**-0.092**

**Table 6:** Averaged model estimates and 95% confidence intervals from the top models predicting the number of fledglings per successful nest (McFadden’s pseudo R^2^ = 0.24).

	No. models	Estimate	SE	Lower CI	Upper CI
NND	1	0.177	0.910	-1.606	1.961
**Farm management**	**2**	**-9.942**	**2.008**	**-13.878**	**-6.005**
Provisioning rate	2	0.084	0.786	-1.457	1.626
**Concealment**	**2**	**9.755**	**1.916**	**6.000**	**13.510**
Provisioning rate x Concealment	1	-0.013	0.241	-0.485	0.459

### Parental provisioning rate

**Fig 1:**
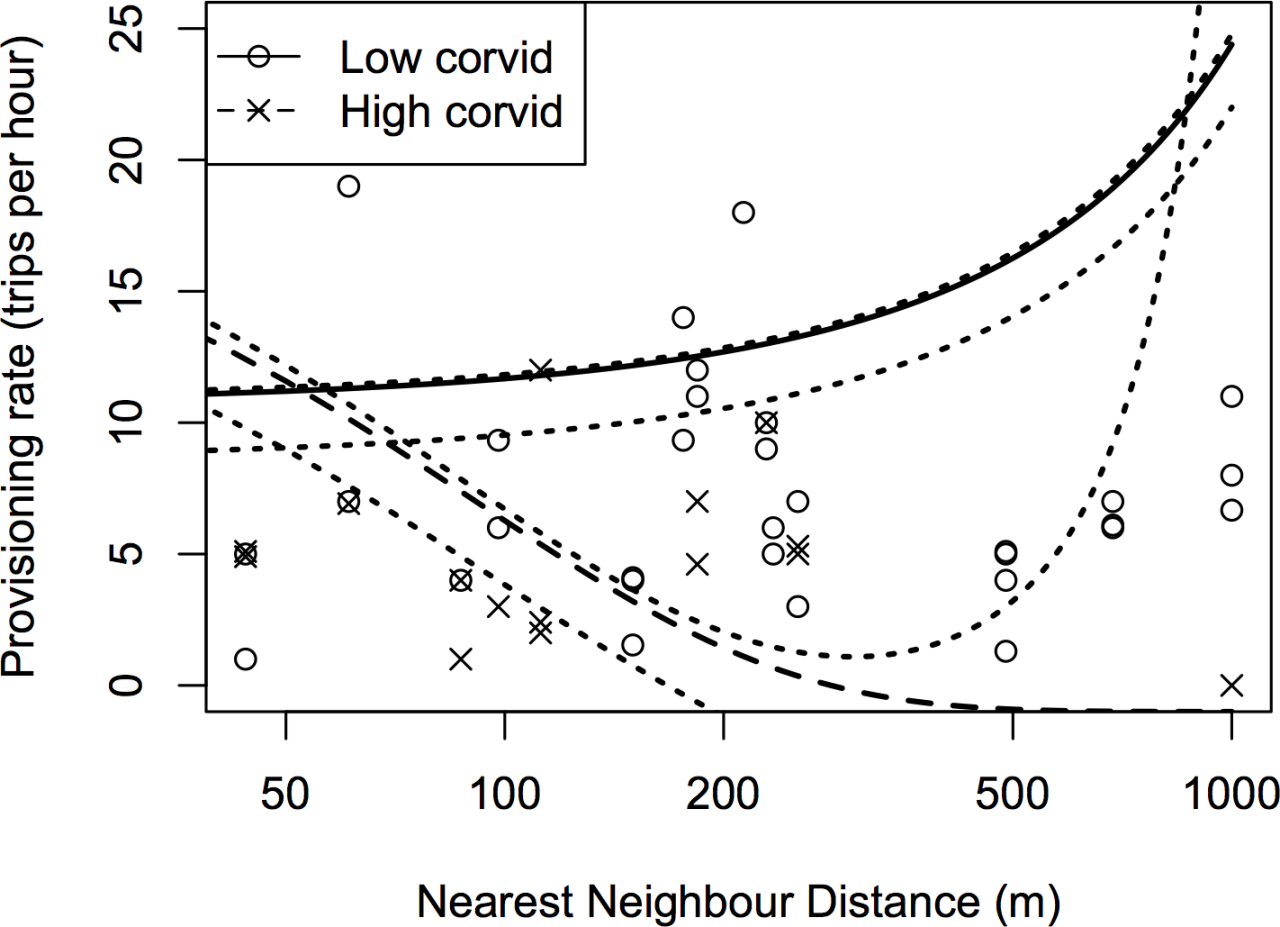
Nestling provisioning rate was influenced by an interaction between NND and corvid abundance. Points show raw data with corvid abundance split into above and below median values (corvid abundance is categorized in the graph for ease of visualization but designated as a continuous variable in the model). Lines are predicted from the final averaged model (Table 7) with median values of other factors causing additional variation in the final model (nestling age = 5 days, brood size = 3 nestlings, temperature = 15°C, year = 2008, farm management = conventional), at the two extremes of observed corvid activity levels along with the median value (low = 0; high = 59; median = 3 corvids passing within 100m of the nest in a 20 minute period prior to assessment of yellowhammer provisioning rate). 95% confidence intervals are shown for all three predictions. For 5 points where provisioning rate was constant for the same nest on more than one occasion, we have added a slight ‘jitter’ (± 0.1 provisioning trip) for display purposes only to allow sample sizes to be visualized.

Parental provisioning rate was assessed on 46 occasions for 17 nests on 12 farms (9 nests on 6 organic farms and 8 nests on 6 conventional farms). Corvid activity and abundance, and invertebrate abundance all differed at the scales at which they were measured (Territory scale: corvid activity: F_29,45_ = 4.139, p<0.001; invertebrate abundance: Linear model (LM), F_194,210_ = 11.55, p<0.001. Farm scale: corvid abundance: LM, F_11,25_ = 6.00, p<0.001; see S1 Table for a summary of territory-scale invertebrate abundance). Provisioning rate was influenced by interactions between territory density (results given from analysis of NND but, as discussed in the methods, throughout similar results arise from NTND) and both corvid activity and brood size (Table 7). At times of low corvid activity, provisioning rates increased with increasing NND (i.e. with decreasing nest density), but at times of high corvid activity, provisioning rate declines with increasing NND (Fig 1). In large broods, provisioning rate declines with increasing NND whereas the opposing trend is seen for small broods. Provisioning rate (trips per hour) was higher on conventionally managed farms than on organic (predicted mean ± SE from final model with median values of all other terms remaining in the final model (Table 7); conventional: 11.55 ± 1.74; organic: 7.58 ± 1.09 trips per hour).

**Table 7:** Averaged model estimates and 95% confidence intervals from the three top models predicting parental provisioning rate (marginal R^2^ = 0.53; conditional R^2^ = 0.54).

Variable	No. models	Estimate	SE	Lower CI	Upper CI
Intercept	3	0.319	0.563	-0.838	1.476
Nestling age	3	0.083	0.043	-0.007	0.172
**Brood size**	**3**	**0.465**	**0.117**	**0.224**	**0.707**
Corvid abundance	3	0.011	0.013	-0.016	0.037
**Farm management**	**3**	**-0.380**	**0.153**	**-0.719**	**0.041**
NND	3	0.003	0.001	-0.001	0.006
Temperature	1	-0.033	0.020	-0.075	0.007
Temperature^2^	1	-0.001	0.001	-0.002	0.001
**Year**	**3**	**0.563**	**0.160**	**0.053**	**1.072**
**Brood size x NND**	**3**	**-0.001**	**0.001**	**-0.001**	**-0.001**
**Corvid abundance x NND**	**3**	**-0.001**	**0.001**	**-0.001**	**-0.001**

### Nestling mass gain

**Fig 2:**
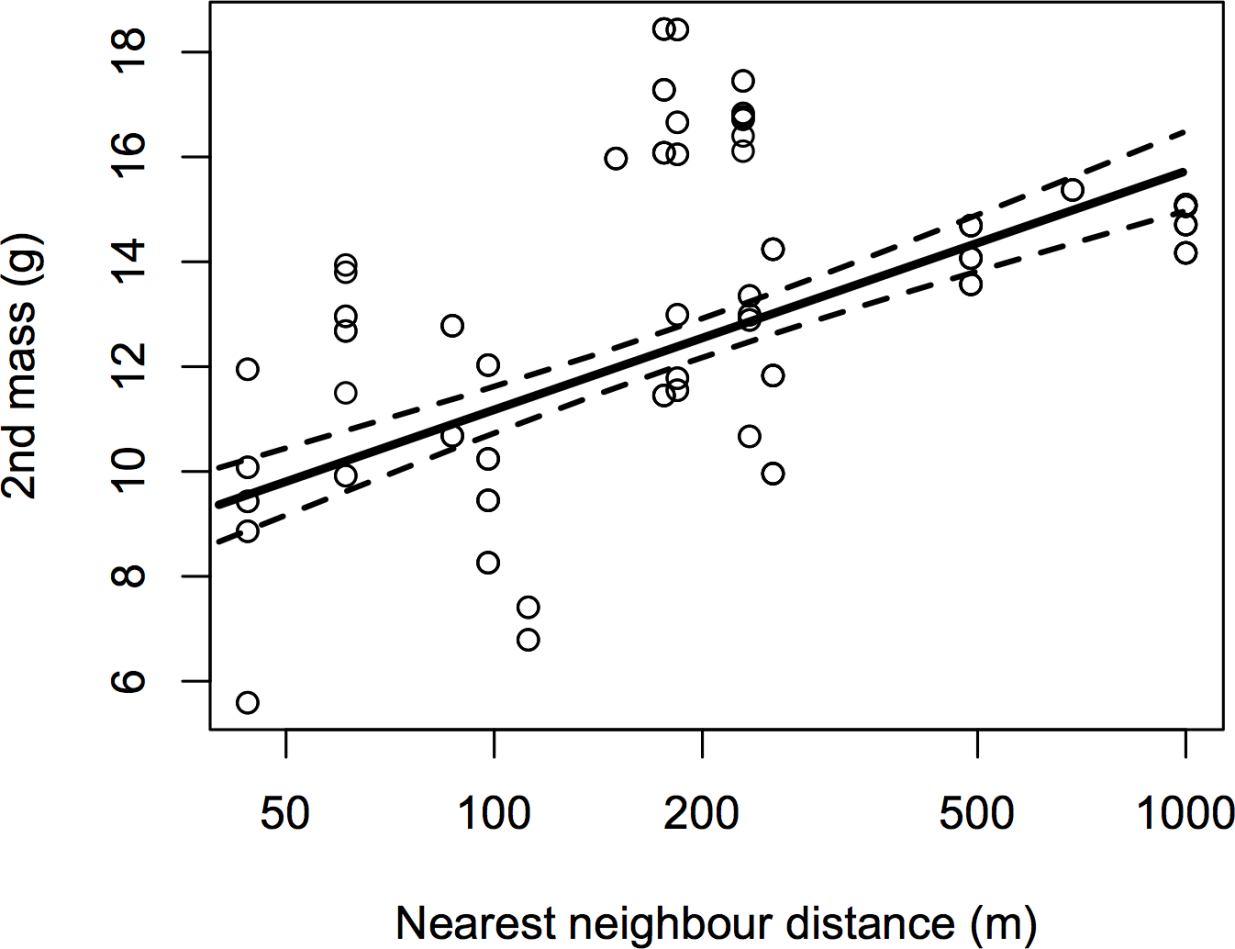
Nestling mass growth increases with increasing NND. Points show raw data; line and 95% CIs are predicted from the final averaged model (Table 8) with median values of other factors retained in the final model (1^st^ mass = 9.3 g, age at 2^nd^ measurement = 5 days, year = 2007, time between measurements = 48 hours, time of 2^nd^ measurement = 15:00)

Nestling mass was assessed for 45 nestlings within 16 nests, of which 42 nestlings within 15 nests were measured on two occasions. NND significantly influenced the magnitude of increase in nestling mass (Table 8), with nestlings in areas of low nesting density gaining mass faster than those in areas of high nesting density (Fig 2)

**Table 8:** Averaged model estimates and 95% confidence intervals from the two top models predicting nestling mass growth rate (Marginal R^2^ = 0.65; Conditional R^2^ = 0.99).

Variable	No. models	Estimate	SE	Lower CI	Upper CI
Intercept	2	-12.895	2.368	-17.606	-8.183
**NND**	**2**	**1.977**	**0.403**	**1.105**	**2.849**
**Hours between measurements**	**2**	**0.064**	**0.010**	**0.045**	**0.083**
1^st^ mass	2	-0.099	0.074	-0.246	0.048
**Age at second measurement**	**2**	**2.583**	**0.298**	**1.990**	**3.175**
Time of second measurement	2	-0.008	0.042	-0.091	0.076
Year	1	0.496	0.782	-1.208	2.200

### Demographic predictions

Given the difference in the number of fledged young between organic and conventional farms, we constructed a simple demographic model to assess the population-level scale of this effect, assuming all other demographic metrics were equal between management types. For 100 pairs of breeding yellowhammers, we used re-nesting estimates calculated by Cornulier *et al*. [49] of 1.66 nests per pair, and a per-nest success rate of 45% from our raw data, to calculate a mean of 0.747 successful nests per pair. Given our model predictions of 2.42 young per nest on organic farms and 3.19 young per nest on conventional farms, this translates into 180.8 young per 100 pairs on organic farms compared to 238.3 on conventional farms. Assuming an adult survival rate of 0.449 and a juvenile survival rate of 0.440 [50], this suggests an annual population change of -15.3% on organic farms compared to -2.7% on conventional farms within our study area.

## Discussion

We investigated whether high breeding densities of animals represent “attractive sinks” within anthropogenically modified environments. Declining species may be more susceptible to such effects [20] and so we used a declining farmland bird as a model. We found that boundary type influenced territory density, with more nesting habitat leading to more territories. Corvid density has increased on farmland over the same timescale as yellowhammer nesting habitat has declined [51], and yellowhammers adjust their behaviour to reduce the likelihood of nest predation by corvids [28]. Given that farmland is a heavily managed environment, we suspected food availability during nestling rearing would not be predictable at the time of territory establishment due to subsequent anthropogenic management. We found that nests in areas of good nesting habitat (i.e. high territory densities) that avoided corvid predation likely suffered increased competition for food, leading to reduced parental provisioning rates and poorer nestling growth in areas of high territory density. Given that farmland is highly anthropogenically influenced there is potential for farm- or landscape management to reduce the magnitude of density dependent effects, which may affect a range of species similarly.

The number of yellowhammer territories was positively associated with the length of available hedgerow. This concurs with previous studies of yellowhammer territory selection that indicate a preference within this species for territories containing suitable nesting habitat [22–24], as the majority of nests are found in hedgerows and dense herbaceous vegetation [30] and hedgerow removal has been linked to the historic declines in yellowhammer populations [26]. The number of territories was also higher within an organic landscape regardless of farm management, supporting findings of wider biodiversity in organic landscapes found by [52], (but see [29]). This confirms our underlying assumption that hedgerow distribution influences territory density [22]. That both hedgerow distribution and territory abundance differ amongst farms confirms our assumption that habitat management amongst farms can influence yellowhammer territory density directly.

Nest concealment was the main factor influencing nesting success, being retained in models of nestling-stage nest failure, brood reduction and the number of fledglings per successful nest. In all cases, well concealed nests were more successful; unsurprising since corvids, considered the main predator of yellowhammer nests [25], are visually oriented predators and are thus more likely to depredate more visible nests [53].

Successful nests on organic farms fledged fewer young than those on conventionally managed farms, translating into a 5 times higher rate of population shrinkage in the absence of immigration. Productivity in our first study year was low due to a wet breeding season, so our overall population trend estimates are likely to be on the low side and our model was designed only to demonstrate the potential for relatively small differences in productivity to translate into a biologically significant difference at the population scale, rather than to provide robust population trend estimates.

Gabriel *et al*. [29] found, on the same study farms, that corvids were associated with organic management at both the farm and landscape scale so it is possible that individuals nesting on organic farms invested less in breeding, laying smaller clutch sizes per breeding attempt [54]. This may be partially compensated through more breeding attempts and our population model assumes re-nesting rates to be constant across farm managements; however, we found no biologically important differences in clutch size (mean ± SE; conventional: 3.15 ± 0.19; organic: 3.55 ± 0.24) between farm managements, or evidence for an influence of management on nest failure through retention of the farm management term in either of our nest failure models. Furthermore, the retention of farm management in the model of brood reduction suggests that brood reduction may be the mechanism behind the reduced number of fledglings on organic farms. Timing of nesting can also influence breeding effort; however all nests considered within our provisioning analyses were found within the range of first egg dates for this species, suggesting that any differences in parental investment are due to other factors.

Competition for food or avoidance of nest predators may reduce the provisioning rate of nestlings, resulting in our findings of a reduction in number of young fledged from successful nests, and a reduction in nestling-stage nest survival. Nestling food resources (arthropods) within our system were more abundant on organic farms [29], and provisioning rate was strongly influenced by an interaction between corvid activity and territory density. This suggests that the association between corvids and organic management found by Gabriel *et al*. [29] leads to corvid activity initially being more important in predicting provisioning rate than food availability. Our data suggest that habitat containing higher yellowhammer territory densities tends to also have higher corvid activity (coefficient ± SE: -0.36 ± 0.12) and abundance (coefficient ± SE: -0.004 ± 0.002). However, when corvid activity was low and thus provisioning was otherwise unrestricted, birds provisioned nestlings at higher rates in areas of low territory density than in areas of higher territory density, although this effect size was larger when comparing low and medium territory densities than when comparing medium and high. This supports the idea of competition for limited food resources in areas providing sufficient nesting habitat to support higher territory densities because each foraging trip will take longer in order to be successful, although we did not assess variation in the size of food load brought back to the nest. When corvid activity was high, the opposite trend is suggested, possibly as birds nesting in more isolated territories may be more obvious to foraging corvids while provisioning nestlings, so requiring behavioural modification to reduce the risk of nest predation [28,55]. The mass gain of nestlings was higher in areas of low territory density, further supporting the idea that density-dependent food limitation occurs where territory density is high.

An alternative scenario is that individual quality influences territory selection and also nesting success. In this situation, high quality birds would defend larger territories in nest locations where they are able to ensure better nest concealment, but lower quality birds would be restricted to nesting in higher densities in less suitable habitat. However, settlement patterns between the two years of our study do not support this suggestion: where territories were lost between years these tended to be in areas of initially low territory density, suggesting a preference within our study sites for areas of high density nesting habitat.

## Conclusions

Overall, our data show a reduced number of fledglings per successful nest on organic farms, which have previously been shown to support higher numbers of corvids as well as to have a higher abundance of nestling food [29]. Whilst our demographic model is simple, it suggests that effects of this size have the potential to alter population dynamics to the extent of forming a population sink. We also found lower parental provisioning rates where corvid abundance was low, and reduced growth where territory density is higher. This suggests the potential presence of an ecological trap whereby preferred nesting habitat does not provide the necessary food resources for optimal reproduction, resulting in a lower breeding output in preferred habitats.

The primary factor determining nestling output appears to be the potential density-independent (lethal) effects of nest predation risk, with higher success rates from more concealed nests, fewer nestlings produced on organic farms where corvid abundance tends to be higher, and a reduction in parental provisioning rate at times of high corvid activity. However, territory density appears to be an important secondary factor with the potential for density-dependent (sub-lethal) effects on nestling quality through restrictions on parental provisioning rates in high territory density areas, along with a corresponding reduction in nestling mass. Our population model suggests the potential for population-level consequences of these lethal effects, but studies have also demonstrated that conditions early in life affect the life-history trajectory of an individual [56,57], with individuals experiencing adverse conditions in the nest suffering a reduced lifespan or body size in adulthood (reviewed in [56,58]). Further work is needed to assess the potential population-level significance of these sub-lethal effects.

Within farmland environments, the opportunity exists for managing populations to maximise breeding success, rather than just breeding densities [59]. Our results suggest complex interactions between farm management, predator abundance, food availability and territory density. We recommend that habitat management for animals within heavily modified environments should focus not just on creating high density nesting habitat, but also on providing sufficient food resources in close proximity to nesting habitat. In particular, boundary habitats such as hedgerows could be managed in order to create a more heterogeneous habitat [60,61], within which populations of socially monogamous, non-colonial animals could be less clustered at the territory scale.

## Supporting Information

S1 Table Summary of territory-scale invertebrate abundance on each of the farms where nests were monitored during the nestling period. Invertebrate abundance is the number of nestling food invertebrates per sample (Hart et al. 2006). Invertebrate abundance differed both between territories (F_192,210_ = 11.546, p<0.001) and between farms (F_199,210_ = 12.582, p<0.001). Data displayed are mean ± 1 SE.
(DOCX)
