# Supplementary material for: Anthropogenically-Mediated Density Dependence in a Declining Farmland Bird
Source: PLoS One. 2015 Oct 2;10(10):e0139492. doi: 10.1371/journal.pone.0139492 (PMC4592266; doi:10.1371/journal.pone.0139492)
Supplement: S1 Table — Invertebrate abundance is the number of nestling food invertebrates per sample (Hart et al. 2006). Invertebrate abundance differed both between territories (F192,210 = 11.546, p<0.001) and between farms (F199,210 = 12.582, p<0.001). Data displayed are mean ± 1 SE. (DOCX) [file pone.0139492.s001.docx]

**Supporting Information**

**S1 Table. Summary of territory-scale invertebrate abundance on each of the farms where nests were monitored during the nestling period.** Invertebrate abundance is the number of nestling food invertebrates per sample (Hart et al. 2006). Invertebrate abundance differed both between territories (F_192,210_=11.546, p<0.001) and between farms (F_199,210_=12.582, p<0.001). Data displayed are mean ± 1 SE.

| **Territory** | **Farm** | **Invertebrate abundance (mean ± 1 SE)** |
| --- | --- | --- |
| 1 | 1 | 102.50 ± 9.36 |
| 2 | 2 | 44.75 ± 6.23 |
| 3 | 3 | 82.92 ± 15.22 |
| 4 | 4 | 99.20 ± 23.25 |
| 5 | 5 | 57.40 ± 10.72 |
| 6 | 6 | 23.00 ± 4.36 |
| 7 | 6 | 76.00 ± 7.57 |
| 8 | 6 | 23.30 ± 4.71 |
| 9 | 7 | 157.00 ± 20.82 |
| 10 | 7 | 87.30 ± 12.41 |
| 11 | 7 | 99.10 ± 12.61 |
| 12 | 8 | 54.42 ± 7.96 |
| 13 | 9 | 87.75 ± 6.52 |
| 14 | 9 | 74.50 ± 11.12 |
| 15 | 10 | 80.50 ± 29.04 |
| 16 | 11 | 190.87 ± 13.50 |
| 17 | 12 | 87.70 ± 11.99 |
